# An Account of Diseases in an Eastern District of London, from July 20 to August 20, 1810

**Published:** 1810-09

**Authors:** 


*An Account of Diseases in an Eastern District of London, from July* 20 *to August* 20, 1810.

| | | | |
|---|---|---|---|
| ACUTE DISEASES. | | Cephalalgia - - - - | 5 |
| Peripneumonia Notha | 5 | Herpes - - - - - - | 4 |
| Cynanche Tonsillaris | 3 | Prurigo - - - - - | 2 |
| Ephemera - - - - - | 4 | Psora - - - - - - - | 1 |
| Rheumatismus Acutus - | 3 | Rheumatismus Chronicus | 9 |
| CHRONIC DISEASES. | | PUERPERAL DISEASES. | |
| Dyspnœa - - - - - | 4 | Ephemera - - - - - - | 5 |
| Tussis - - - - - - - | 6 | Menorrhagia Lochialis - | 4 |
| Tussis cum Dyspnœa - | 3 | Mastodynia - - - - | 6 |
| Hæmoptysis - - - - | 2 | Stranguria - - - - - | 3 |
| Phthisis Pulmonalis - - | 2 | INFANTILE DISEASES. | |
| Raucedo - - - - - - | 3 | Erysipelas Infantile - - | 2 |
| Gastrodynia - - - - | 5 | Vermes - - - - - - - | 4 |
| Hæmorrhois - - - - | 3 | Convulsio - - - - - - | 2 |
| Scrophula - - - - - | 4 | Aphthæ - - - - - - | 3 |
| Hypochondriasis - - - | 2 | | |

Several instances of cutaneous affections have lately occurred both in children and adults, some of which have proved very tedious and obstinate.

This disease is in some cases merely local; it is not preceded by any indisposition, nor does it produce any other unpleasant sensation. Under these circumstances it will often yield to some external application. But in other instances these unpleasant appearances on the surface depend upon some morbid state of the constitution, which requires the immediate attention of the practitioner; and before any attempt is made to remove the complaint by external means, the general habit must be improved, and the original disease, of which the other is perhaps only the symptom, must be attended to. It has indeed too often been discovered, that an impatience to get rid of what is is unpleasant in appearance, though sometimes salutary in its effects, has been productive of the most serious consequences. From the high character which has been publickly given to some medicines, or the recommendation of friends, who have been relieved from what they falsely suppose a similar disease, experiments have been made, which, if they have succeeded in removing the symptom, have entailed upon the patient a disease of the most serious nature, and which has sometimes baffled every attempt made by the most skilful practitioner to remove.

